# Incremental Theory of Intelligence Moderated the Relationship between Prior Achievement and School Engagement in Chinese High School Students

**DOI:** 10.3389/fpsyg.2017.01703

**Published:** 2017-09-27

**Authors:** Ping Li, Nan Zhou, Yuchi Zhang, Qing Xiong, Ruihong Nie, Xiaoyi Fang

**Affiliations:** Institute of Developmental Psychology, Beijing Normal University, Beijing, China

**Keywords:** prior achievement, school engagement, theory of intelligence, Chinese high school students

## Abstract

School engagement plays a prominent role in promoting academic accomplishments. In contrast to the relative wealth of research that examined the impact of students’ school engagement on their academic achievement, considerably less research has investigated the effect of high school students’ prior achievement on their school engagement. The present study examined the relationship between prior achievement and school engagement among Chinese high school students. Based on the Dweck’s social-cognitive theory of motivation, we further examined the moderating effect of students’ theories of intelligence (TOIs) on this relationship. A total of 4036 (2066 girls) students from five public high school enrolled in grades 10 reported their high school entrance exam achievement in Chinese, Math and English, school engagement, and TOIs. Results showed that (a) students’ prior achievement predicted their behavioral, emotional, and cognitive engagement, respectively, and (b) the association between prior achievement and behavioral, emotional, and cognitive engagement is strong for students with an incremental theory but not for those with an entity theory in the emotional and cognitive engagement. These findings suggest that prior achievement and incremental theory were implicated in relation to adolescents’ school engagement. Implications and future research directions were discussed.

## Introduction

Over the past decade, researchers on adolescents’ academic development have become increasingly interested in investigating the Chinese students’ learning and achieving (Hau and Ho, 2010). One of the reason is that Chinese education leads the world in comparisons of educational achievement. For example, the results from the cross-national Program for International Student Assessment (PISA, Gurria, 2014) consistently rank Shanghai-China as first in mathematics, reading, and science performance. Another reason is that Chinese high school and university admissions are almost solely determined by students’ academic achievement (i.e., test scores in entrance exams), students who attain higher scores go to key high schools and universities, while others go to regular high schools and universities (Hu et al., 2015). Promoting educational success is also a key policy objective of the Chinese government (e.g., Ministry of Education of the People’s Republic of China, 2010).

One promising avenue for improving Chinese adolescents’ educational prospects is school engagement, or the degree to which students are involved in and committed to the academic and social activities in school, as such engagement plays an influential role in promoting academic success and influencing a wide range of adolescent outcomes (see Upadyaya and Salmela-Aro, 2013, for a review). A large number of studies have examined the unidirectional predictive relationship between school engagement and academic achievement (e.g., Li and Lerner, 2011, 2013), such as students who are actively engaged in school are more likely to perform well academically (Abbott-Chapman et al., 2014). Little research, however, has focused on the opposite direction of effects, that is, the impact of prior achievement on school engagement. Indeed, the prior achievement is one of a very important antecedent variable that predict school outcomes (Hattie, 2009). Previous study has found that the associations between achievement and engagement appear to vary by students’ theories of intelligence (i.e., beliefs about whether intelligence is fixed or malleable, TOIs) (e.g., Hong et al., 1999). In the current study, we invested the links between prior achievement and school engagement. We placed particular attention on the extent to which students’ TOIs conditioned the primary relations of interest. That is, we sought to determine whether the links between prior achievement and school engagement were stronger or weaker for students with an incremental versus students with an entity theory. Such work can elucidate the value of educational interventions targeting students’ TOIs and suggest the extent to which such efforts could be more or less effective if targeted to specific student populations.

### Prior Achievement and School Engagement

Most researchers view school engagement as a multidimensional construct, including behavioral, emotional, and cognitive components (Fredricks et al., 2004; Reschly and Christenson, 2012; Li and Lerner, 2013). Behavioral engagement refers to the range of actions that reflect participation in school-based activities; emotional engagement refers to students’ affective reactions (i.e., interest, boredom, or anxiety) in the classroom and toward school; and cognitive engagement refers to students’ investment (i.e., motivation, strategic learning skills, and problem solving) in learning (Fredricks et al., 2004; Upadyaya and Salmela-Aro, 2013; Owen et al., 2016). These components describe different aspects of school engagement and are positively associated with each other (Fredricks et al., 2004; Li and Lerner, 2013).

Previous studies have documented the effects of school engagement on adolescents’ academic achievement (e.g., Wang and Holcombe, 2010; Wang and Peck, 2013; Wang et al., 2015). For instance, Scheidler (2012) found that a tripartite measure of school engagement positively predicted standardized test scores among 8th Grade students. The identification of this association adds to our understanding of school engagement but provides little information about three important issues. Firstly, the previous work on school engagement has been empirically tested by using Western participants. Although the total number of Chinese high school students in 2015 was 23 million (National Bureau of Statistics of China, 2016), representing the largest group in the world, little is known about the Chinese high school students’ school engagement. Secondly, previous studies have overwhelmingly emphasized the unidirectional effects of school engagement on academic achievement, little is known about the effect of prior achievement on school engagement. Indeed, students’ prior achievement (i.e., performance on standardized tests) has been widely viewed as a strong predictor of their success in school (Kitsantas and Zimmerman, 2009). Researchers also found that prior achievement is associated with some aspects of school engagement (e.g., Garavalia and Gredler, 2002; Liem et al., 2008), such as students with low prior achievement often avoid seeking help when struggling (Du et al., 2016).

Thirdly, recent research has examined the reciprocal relationship between school engagement and academic achievement among high school years (i.e., grades 10, 11, and 12). The results showed that school engagement and academic achievement were mutually predictive and that these predictions varied from grade to grade (Chase et al., 2014). However, to our knowledge, no existing study has investigated the relations between prior achievement and school engagement over the high school transition period (i.e., new 10th graders). These new 10th graders represent a large population of Chinese high school students—approximately eight million individuals in 2015 year (National Bureau of Statistics of China, 2016). When they make the transit into high schools, they are faced with greater academic stress than their Western counterparts at this stage because of the fierce competition of college entrance examination, which is usually considered to be the most important examination for all Chinese students (Liu and Lu, 2011a,b). They also experience an exceptional amount of pressure from teachers and parents to succeed in college entrance exams (Li and Prevatt, 2008).

### TOIs as a Moderator

According to Dweck’s (2000) social-cognitive theory of motivation, students may hold different beliefs about the nature of intelligence (i.e., whether intelligence is fixed or malleable). Some students believe it to be more of a fixed unchanging “entity,” while other students regard it as something that is malleable—something that can be improved or increased with effort. Well-documented differences exist in the links between students’ TOIs and both academic achievement and school engagement. Empirical research suggests that the two beliefs of intelligence shape students’ achievement in different ways such that an incremental theory of intelligence facilitates and an entity theory of intelligence inhibits students’ achievement (e.g., Good et al., 2003; Blackwell et al., 2007; Paunesku et al., 2015). Similarly, some evidence suggested that TOIs may be a key factor in explaining some aspects of school engagement, wherein students with an entity theory showed more helplessness approach to schoolwork, increased academic self-handicapping, truancy, and disengagement, and are more likely to feel various types of negative emotions such as anger, anxiety, shame, hopelessness, and boredom (King et al., 2012; Wang and Ng, 2012; De Castella and Byrne, 2015).

In addition to these well-established differences, there is some evidence that the relations between prior achievement and school engagement may be conditioned by TOIs. For example, students with an incremental theory were more likely to believe that working hard was necessary and effective in achievement than those students with entity theory (Blackwell et al., 2007). Students with an incremental theory were also show greater engagement, persistence, and resilience in the face of learning setbacks (Mangels et al., 2006) and were more likely to take remedial action aimed at improving future achievement (Hong et al., 1999). Most relevant to the present study, previous studies suggested that low prior achievement students’ help-seeking avoidance is likely underscored by academic self-efficacy. That is, students with low self-efficacy are more likely to believe that the need for help is confirmation of low ability, therefore, they are less likely to seek help. In contrast, students with high self-efficacy do not worry that the need for help indicates that their lack of ability and are more likely to secure the necessary help (Ryan and Shin, 2011; Amemiya and Wang, 2016). Based on these findings, we expected that students’ TOIs would condition the association between prior achievement and school engagement. Specifically, students with an incremental theory may be more likely to view their prior achievement as a diagnosis of the engagement they have engaged in school and thus may continue to engage in schoolwork to improve their abilities. In contrast, students with an entity theory often consider their prior achievement as largely a diagnosis of their innate abilities and might be less engaged in schoolwork given their view of ability as innate rather than dynamic.

### The Current Study

The main goals of present study were twofold. The first goal was to examine the associations between Chinese high school students’ prior achievement and their school engagement. Based on the extant literature, we hypothesized that students’ prior achievement will be positively related to school engagement. The second goal was to explore whether TOIs had an impact on the relationship between prior achievement and school engagement. In this respect, we hypothesized that prior achievement was more strongly associated with school engagement among students with an incremental theory than among those with an entity theory.

Notably, prior research has demonstrated that school engagement was influenced by family socioeconomic status (SES), gender, and educational expectations (Li et al., 2010; Li and Lerner, 2011). Thus, in order to eliminate the effects of the variables stated above, the present study considered these variables as control variables.

## Materials and Methods

### Participants

The participants consisted of 4036 (2066 girls) students (mean age = 15.41, *SD* = 0.55) enrolled in grades 10. These students were recruited form five public high school in Panyu District of Guangzhou, located in Guangdong province, southern China. Three of them are key high schools (3107 students), and the rest two are regular high schools (929 students).

### Procedure

As part of a larger study, the students filled in the questionnaires during the first semester. All participants were informed about the confidentiality and anonymity of their responses and were encouraged to provide honest answers in the questionnaire. Also, students were assured that they could quit the session whenever they decided to do so. The survey took place in the classroom in groups of 24–55 students, on a regular school day, in the presence of an experienced research assistant, and lasted for about 40 min. Permission for the study was obtained from school authorities and principals. The oral consent forms were obtained from students and their parents.

Several demographic questions were asked about students, including age, gender, educational expectations, parents’ marital status, health status, and family SES.

### Measures

#### Prior Achievement

Students’ high school entrance exam achievement in Chinese, Math, and English were obtained from the school. The entrance exam took place in 3 months before our survey. Scores for these three major subjects have been found to be a valid measure of school academic achievement in China (Chen et al., 2011). Scores in each subject were standardized within the grade. The mean across the three subjects was taken, with higher scores representing higher achievement (Zhao and Wang, 2014).

#### School Engagement

We used the 15-item Behavioral–Emotional–Cognitive School Engagement Scale (BEC-SES) to measure the tripartite school engagement (Li, 2010). The behavioral engagement subscale includes five items indicating contribution to class discussion, preparation, skipping class, and finishing homework on time. The response format ranged from 1 (never) to 4 (Always). The Cronbach’s alphas in the current study was 0.56. The emotional engagement subscale includes five items that assess students’ sense of belonging and affect toward school. The response format ranged from 1 (strongly disagree) to 4 (strongly agree). The Cronbach’s alphas in the current study was 0.74. The cognitive engagement was measured by five items designed to assess the extent to which students valued education and things learned at school, as well as their thoughts about learning. The response format was also a 4-point Likert scale, ranging from 1 strongly disagree) to 4 (strongly agree). The Cronbach’s alphas in the current study was 0.89.

#### Theories of intelligences

Three items were adopted from the Implicit Theory of Intelligence Scale (e.g., Hong et al., 1999) to measure participants’ entity theory of intelligence (“You have a certain amount of intelligence and really cannot do much to change it,” “Your intelligence is something about you that you can’t change very much,” and “You can learn new things, but you can’t really change your basic intelligence”). All items were scored on a 6-point Likert-type scale ranging from 1 (strongly disagree) to 6 (strongly agree). The responses were reverse coded; the mean of three items was taken, with higher scores indicating a stronger incremental theory of intelligence (e.g., Dweck et al., 1995). This measure has acceptable reliability and validity in China (e.g., Wang and Ng, 2012; Zhao and Wang, 2014). In the current study, the alpha coefficient for the measure was 0.94.

#### SES and Educational Expectations

We used the information concerning parents’ educational level and current occupations to assess family SES (Yang et al., 2010). Specifically, paternal/maternal education level consisted of five categories: (1) elementary school, (2) junior high school, (3) senior high school (including occupational middle school), (4) up-to-3-year college, and (5) 4-or-more-year university. Occupation level consisted of three categories: no job, part-time job, and full-time job. These two indicators were all treated as ordinal scale. A composite SES score was then created by standardizing educational and occupational levels (*Z*-score) and adding the standardized scores (Janicki-Deverts et al., 2007).

Educational expectations were assessed by asking students what final academic degree they wanted to obtain (i.e., 1 = high school, 2 = junior college, 3 = undergraduate, 4 = master, and 5 = doctor), with higher scores corresponding to higher educational expectations.

### Analytic Procedures

Hypotheses were evaluated by conducting path analysis with Mplus version 7.4 (Muthén and Muthén, 2012). Given that the tested models were just identified (i.e., number of identifying restrictions equaled number of estimated parameters), no indices of global fit are reported. Missing values (ranged from 0 to 0.50% on grade) were addressed using full information maximum likelihood estimation methods, a preferred technique for producing parameter estimates with minimal bias (Schlomer et al., 2010).

## Results

The descriptive statistics for the study variables are shown in **Table 1**. The correlations among prior achievement, TOIs, and three dimensions of school engagement were statistically significant and in the expected directions.

**Table 1:** Correlations and descriptive statistic among variables.

	1	2	3	4	5	6	7	8	9	10	11	*M*	*SD*
1. School type													
2. Gender	0.05**												
3. Age	-0.08**	-0.06**										15.41	0.55
4. Health status	0.03*	-0.09**	0.01									4.07	0.83
5. Educational expectations	0.19**	-0.09**	-0.01	0.11**								3.60	0.90
6. Parents’ marital status	-0.02	0.01	-0.01	-0.09**	0.02							1.27	0.89
7. SES	0.21**	-0.03*	-0.05**	0.11**	0.20**	-0.09**						0.00	0.64
8. Prior achievement	0.61**	0.10**	-0.06**	0.01	0.26**	-0.03	0.22**					0.00	11.34
9. Behavioral engagement	0.11**	0.15**	-0.02	0.19**	0.11**	0.02	0.06**	0.17**				3.10	0.53
10. Emotional engagement	0.18**	0.09**	0.01	0.25**	0.16**	-0.01	0.09**	0.17**	0.60**			3.01	0.58
11. Cognitive engagement	0.10**	0.10**	0.03	0.20**	0.16**	-0.03	0.07**	0.14**	0.50**	0.69**		3.16	0.66
12. TOIs	0.06**	-0.02	-0.01	0.06**	0.15**	0.01	0.04**	0.10**	0.24**	0.16**	0.06**	4.10	1.33


^∗^*p* < 0.05, ^∗∗^*p* < 0.01, ^∗∗∗^*p* < 0.001.

**FIGURE 1:**
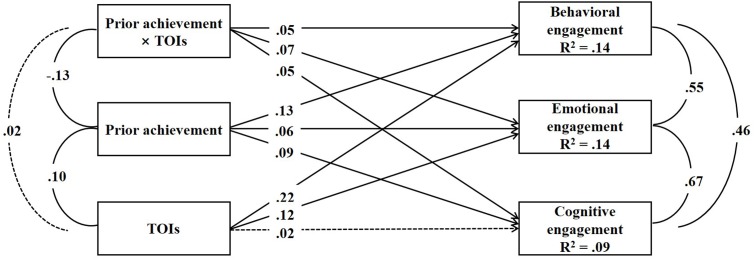
Associations among prior achievement, TOIs, and behavioral, emotional, and cognitive engagement. *N* = 4036. Solid lines indicated significant associations. Dotted lines indicated non-significant associations. Standardized coefficients were presented.

Results for the model are presented in **Figure 1**. Prior achievement, TOIs, and the interaction between prior achievement and TOIs were specified as predictors of behavioral, emotional, and cognitive engagement. The school, gender, age, health status, educational expectations, SES, and their parents’ marital status were specified as control variables linked to behavioral, emotional, and cognitive engagement. Consistent with hypotheses, prior achievement was positively associated with behavioral, emotional, and cognitive engagement, respectively. Associations between prior achievement and behavioral, emotional, and cognitive engagement were not different from each other using a ward test [behavioral versus emotional: 95% CI (-0.002, 0.001); behavioral versus cognitive: 95% CI (-0.002, 0.002); cognitive versus emotional: 95% CI (-0.002, 0.001)].

**FIGURE 2:**
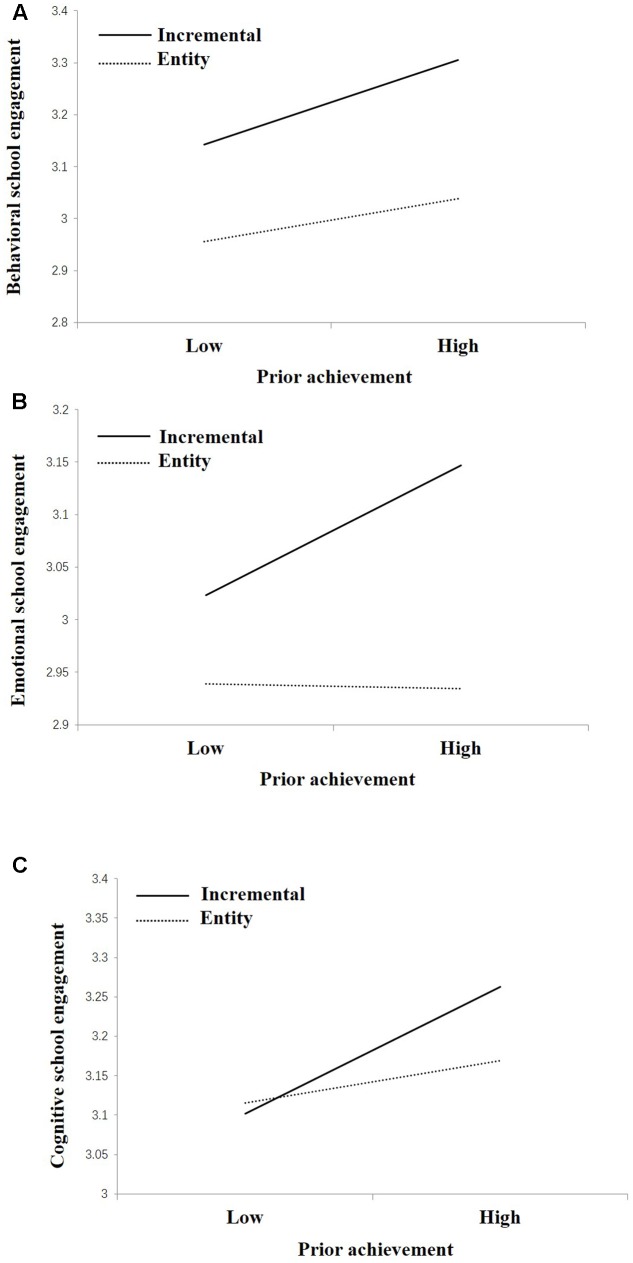
Prior achievement interacted with TOIs in the prediction of **(A)** behavioral school engagement, **(B)** emotional school engagement, and **(C)** cognitive school engagement.

Prior achievement also interacted with TOIs in the prediction of behavioral, emotional, and cognitive engagement, respectively (**Figure 2**). Specifically, the association between prior achievement and behavioral engagement is stronger for students with an incremental theory (*b* = 0.01, β = 0.17, *p* < 0.01) than those with an entity theory (*b* = 0.004, β = 0.08, *p* < 0.01). Prior achievement was associated with emotional engagement for students with an incremental theory (*b* = 0.01, β = 0.12, *p* < 0.01) but was not associated with emotional engagement for students with an entity theory (*b* = -0.0002, β = -0.004, *p* > 0.05). Similarly, prior achievement was associated with cognitive engagement for students with an incremental theory (*b* = 0.01, β = 0.13, *p* < 0.01) but was not associated with cognitive engagement for students with an entity theory (*b* = 0.003, β = 0.04, *p* > 0.05).

## Discussion

School engagement plays a prominent role in aspects of academic success and individual well-being (Upadyaya and Salmela-Aro, 2013). The present study examined the relationship between Chinese high school students’ prior achievement and their school engagement, and the moderating effect of students’ TOIs on this relationship. We found that prior achievement significantly predicted school engagement. More importantly, students’ TOIs moderated the relationship between prior achievement and school engagement.

In consideration of school engagement is presumed to be malleable (Fredricks et al., 2004), recent research has explored predictors of school engagement (e.g., Hospel and Galand, 2016). For example, Dotterer and Lowe (2011) found that classroom context is an important predictor of school engagement. With some important exceptions (Chase et al., 2015), the present study explored the effect of prior achievement on school engagement. Consistent with the findings in the Western societies (Chase et al., 2014), we found that Chinese high school student’s prior achievement significantly predicted their behavioral, emotional, and cognitive school engagement. These findings support the notion that academic achievement is not only an outcome, but can also predict the degree to which students are engaged in school. That is to say, the more that students thrive in school academically, the more efficacious they feel, which, in turn, increases all three components of engagement in the school context (Salmela-Aro and Upadaya, 2012; Chase et al., 2014). This high relationship accounts for what many researchers call the “Matthew effect,” which is based on the biblical notion that rich get richer and the poorer get poorer or do not gain as much (Hattie, 2009).

We found that Chinese students’ TOIs moderated the relationship between prior achievement and behavioral, emotional, and cognitive engagement. The association between prior achievement and behavioral, emotional, and cognitive engagement is strong for students with an incremental theory but not for those with an entity theory in the emotional and cognitive engagement. Such result can largely be attributed to differences in incremental and entity students’ beliefs about the role of effort in achievement (Miele and Molden, 2010; Miele et al., 2011; De Castella and Byrne, 2015; see also Yeager and Dweck, 2012, for a review). The effort dimension is a key element for distinguishing between the incremental versus entity theory of intelligence. Specifically, students with an incremental theory attribute their academic achievement to effort more often than to ability, whereas students with an entity theory attribute their academic achievement to innate and stable ability rather than to effort (e.g., Blackwell et al., 2007; Miele et al., 2011). Although effort is perceived as a tool to improve ability in an incremental theory of intelligence, it is evidence of a lack of ability in an entity theory of intelligence (Blackwell et al., 2007). As a result, students with an incremental (versus entity) theory were more likely to take remedial action if performance was unsatisfactory (Hong et al., 1999), and more likely to make school engagement, such as seek out help from their teacher and take more advanced math course (Romero et al., 2014). It is also argued that Chinese students typically do not regard intelligence as fixed but malleable through learning, enabling them to take a persistent rather than helpless approach to schoolwork, and subsequently perform well (Hau and Ho, 2010).

Moreover, effort-making is regarded as a necessary means to improve one’s learning, especially in Chinese context. As some popular Chinese proverbs describe, “making effort to compensate for inadequate intelligence (qín néng bú zhuo),” “With persistence, an iron pestle can be ground down to a needle (tiě chu mó chéng zhcn),” “Learning is like rowing upstream; not to advance is to drop back (xúe rú nì shuǐ xíng zhoū, bù jìn zé tuì),” “practice makes perfect (shú néng shēng qiǎo).” These beliefs are the reasons why many Chinese parents and teachers constantly encourage their children or students to invest full school engagement in academic learning, even if the students are already performing well (Li, 2012; Chen et al., 2016).

Another possible explanation involved goal setting (Burnette et al., 2013). Previous research has shown that students with an entity theory are more concerned with pursuing performance goals (i.e., goals aimed at documenting their ability) (Cury et al., 2006). As a consequence, these students are more likely to shun opportunities (i.e., behavior, emotional, and cognitive engagement in the present study) for learning, to give up easily when faced with challenges or setbacks, and generally to avoid tasks they might have difficulties to master (Hong et al., 1999; Dupeyrat and Mariné, 2005). Moreover, students with an entity theory believe they succeed because they are lucky, not because they are exert a lot of effort (Robins and Pals, 2002). Conversely, Chinese students with an incremental theory pursue learning and mastery goals (i.e., goals aimed at increasing their ability) (Chen and Wong, 2015). These students are more likely to think that school engagement is necessary and effective for achieving goals; they also show more willingness to engage effort and to change strategy when facing failure as well as success (i.e., low and high prior achievement) than students with an entity theory (Robins and Pals, 2002; Blackwell et al., 2007; Chen and Wong, 2015). Moreover, students with an incremental theory view their success as a result of engagement, they do not believe luck plays an important role in their success (Robins and Pals, 2002).

Prior achievement was not associated with emotional and cognitive engagement for students with an entity theory. A possible explanation about this observation is that emotional and cognitive engagement (versus behavioral engagement) are not explicitly or directly related to the school-based activities (i.e., prior achievement) since these two factors are internalizing behaviors and can be attained outside the school environment. Consistent with this explanation, previous studies found that the regression coefficient for emotional engagement on achievement was not as strong as behavioral engagement on achievement (Chase et al., 2014). An alternative explanation would be that high school students with an entity theory do not want to change their emotional and cognitive engagement. As suggested by Heckman and Masterov (2007), by the time students have reached high school, their study habits and attitudes toward school are entrenched to such a degree that very little will alter their academic achievement. In the current study, this phenomenon of stable attitudes and performance may have manifested itself especially for those students with an entity theory. Previous studies supported this explanation as well, such that the predicted effects of entity theory of intelligence on emotional (i.e., enjoyment, hope, and pride) and cognitive (i.e., deep and shallow strategy use) engagement in learning failed to emerge (Dupeyrat and Mariné, 2005; King et al., 2012).

In sum, the current study contributed to the research on students’ school engagement in at least three ways. First, we contributed to the study of Chinese high school students’ school engagement by using a multidimensional measure, including behavioral, emotional, and cognitive engagement. It has been able to provide a much richer picture of this dynamic concept, as well as a fully explanation of students’ behavior and attitudes toward school. Second, we built upon previous research by exploring antecedents of school engagement by testing whether prior achievement predicted school engagement. Third, we built upon previous research by examining whether TOIs moderated the link between prior achievement and school engagement.

Despite all this, our study is not without limitations. Chase et al. (2014) suggested that the strength of the association between school engagement and academic achievement has been found to vary significantly depending on how engagement is measured, such as self-reported or observational assessments. Future studies may consider a triangulation of measures from both observational assessments (i.e., teacher and parent ratings) and students (i.e., in the current study) in order to evaluate these constructs in adolescence more effectively. The use of multiple methods can also reduce mono-reporter bias and inflated responses due to social desirability. In addition, the present study is a correlational study, which could not establish a causal relationship. Considering that previous study suggested that TOIs as a point of intervention in efforts to enhance students’ academic success (Yeager and Dweck, 2012), further work might implement a growth mindset intervention with high school students and determine its impact on the relationship between academic achievement and school engagement. Specifically, future research should focus on improving students’ incremental theory to address the inadequate school engagement of low prior achievement students over the high school transition period (Yeager et al., 2016).

## Ethics Statement

This study was carried out in accordance with the recommendations of Ethics Review Committee at the School of Psychology, BNU with written informed consent from all subjects. All subjects gave written informed consent in accordance with the Declaration of Helsinki. The protocol was approved by the Ethics Review Committee at the School of Psychology, BNU.

## Author Contributions

PL, NZ, and XF designed this study and draft the manuscript; PL, YZ, QX, and RN performed research; PL, NZ, and XF analyzed data. All authors approved the final version of manuscript for submission.

## Conflict of Interest Statement

The authors declare that the research was conducted in the absence of any commercial or financial relationships that could be construed as a potential conflict of interest.
